# Safety and efficacy analysis of the off-label use of pipeline embolization devices for intracranial aneurysms: a propensity score matching study

**DOI:** 10.3389/fneur.2023.1278366

**Published:** 2024-01-04

**Authors:** Yajing Ma, Xin Deng, Zhen Chen, Yongjie Yuan, Sheng Guan, Xinbin Guo

**Affiliations:** ^1^Department of Interventional Neuroradiology, The First Affiliated Hospital of Zhengzhou University, Zhengzhou, Henan, China; ^2^Neurointervention Engineering Research Center of Henan Province, Zhengzhou, Henan, China

**Keywords:** pipeline embolization device, off-label, propensity score matching, intracranial aneurysm, pipeline

## Abstract

**Background and objective:**

The safety and efficacy of on-label use of pipeline embolization devices (PEDs) are well established; however, there is much controversy over their off-label use. This study aimed to investigate the safety and efficacy of the off-label use of PEDs for treating intracranial aneurysms.

**Methods:**

This single-center study retrospectively included patients with digital subtraction angiography, computed tomographic angiography, or magnetic resonance angiography confirmed intracranial aneurysms treated with PEDs who were admitted to our institution between 1 January 2018 and 1 July 2022. Patients were divided into on- and off-label groups according to the Food and Drug Administration criteria published in 2021. Propensity score matching (PSM) was used to balance disparities in baseline information between the two groups. Safety outcomes included postoperative mortality and complication rates, whereas effectiveness outcomes included aneurysm occlusion rate (O’Kelly-Marotta grading system C + D grades), retreatment rate within 12 months, and postoperative functional score [modified Rankin scale (mRS) score]. The study was approved by the Ethics Committee of Scientific Research and Clinical Trial of the First Affiliated Hospital of Zhengzhou University (Ethics number: KY 2018–098-02). All patients provided informed consent.

**Results:**

A total of 242 patients with 261 aneurysms (160 on-label and 101 off-label aneurysms) were included in this study. PSM yielded 81 pairs of patients matched for baseline information. Postoperative hemorrhagic, ischemic, and procedure-related complication rates did not reach statistical significance. In addition, no statistically significant differences in the aneurysm occlusion rate, retreatment rate within 12 months, postoperative functional score (mRS score), or mRS score deterioration rate were observed between the two groups. A higher incidence of in-stent stenosis was observed in the off-label (4.9% vs. 21%, *p* = 0.002) group than in the on-label group; however, all patients were asymptomatic.

**Conclusion:**

Compared with on-label use, off-label use of PEDs for treating intracranial aneurysms did not increase the risk of complications, and the occlusion rates were comparable. Therefore, decisions regarding clinical management should not rely solely on on- or off-label indications.

## 1 Introduction

Pipeline embolization devices (PEDs) were the first flow-directed devices approved for treating intracranial aneurysms with a higher metal coverage rate and mesh density than conventional stents. PEDs play an immediate protective role against aneurysms by altering hemodynamic factors. Furthermore, owing to their higher metal coverage rate, they support endothelial growth, facilitating intima formation within the stent and promoting late occlusion (1, 2). Based on the Pipeline for Uncoilable or Failed Aneurysms (PUFS) trial, the Food and Drug Administration (FDA) approved the indication of PEDs for “adult patients with unruptured, large or giant, wide-necked intracranial aneurysms from the petrous to superior hypophyseal segments of the internal carotid artery (ICA)” in 2011 (3). Based on the Prospective Study on Embolization of Intracranial Aneurysms With the Pipeline Embolization Device (PREMIER), the indication was expanded in 2018 to include fusiform aneurysms and saccular aneurysms with a neck greater than 4 mm up to the ICA terminus (4). The on-label use of PEDs has been proven safe and effective. However, current surgical treatment options are technically challenging and have high complication rates for the off-label treatment of posterior circulation, distal circulation, bifurcation, previously treated, acutely ruptured, and other complex aneurysms with no practical clinical remedy. Moreover, the FDA has yet to include complex aneurysms, such as blisters, ruptured, distal anterior circulation, and posterior circulation aneurysms, as indications for PED treatment. In this study, we aimed to share our experience of treating intracranial aneurysms using PEDs and explore their possible off-label use.

## 2 Methods

The study was approved by the Ethics Committee of Scientific Research and Clinical Trial (Ethics number: KY 2018–098-02). All patients provided informed consent.

### 2.1 Data collection and definitions

The inclusion criteria were as follows: (1) intracranial aneurysms confirmed using digital subtraction angiography (DSA), magnetic resonance angiography (MRA), or computed tomographic angiography (CTA); (2) treatment with PEDs for intracranial aneurysms during the research period; and (3) complete clinical and imaging follow-up data.

The exclusion criteria were as follows: (1) significant vascular tortuosity and difficult-to-measure relevant data; (2) poor compliance and failure to take medication according to prescriptions; (3) combination of other organic abnormalities and failure to trace clinical symptoms; and (4) incomplete clinical and imaging data.

Regarding grouping standards, the on-label use of PEDs approved by the FDA in 2018 (5) is applied when: (1) patients are aged ≥22 years; (2) aneurysms are located in the internal carotid artery; (3) neck–body ratio is >0.5 or aneurysm neck is >4 mm; (4) aneurysm morphology is fusiform or saccular; (5) aneurysm is non-ruptured (acute rupture defined as ≤60 days); (6) the target vessel’s diameter is 2–5 mm; and (7) there is no history of stent placement at the site where the PED is to be implanted. Failure to meet any of the above criteria is considered to be an off-label aneurysm.

The following information was collected: (1) baseline data including sex, age, major comorbidities, preoperative modified Rankin scale (mRS) score, and others, (2) aneurysm-related data including location, neck, maximum diameter, diameter of the proximal and distal aneurysm-carrying arteries, whether the aneurysm was multiple, ruptured, and incorporated with a branch, history of stent placement at the target aneurysm site, and immediate postoperative and follow-up imaging grading using OKM grading criteria (6), (3) perioperative complications (procedure-related, ischemic complications, and hemorrhagic complications) and complication rates during follow-up, and (4) mRS scores, which were used as reference points to rate patients’ neurological functions. An mRS score ≤ 2 indicated a good prognosis, whereas an mRS score ≥ 3 indicated a poor prognosis. The primary outcome was the safety of PED off-label application, i.e., postoperative patient mortality and complication rates, whereas secondary outcomes were the effectiveness of PED off-label application, i.e., aneurysm occlusion rates (O’Kelly-Marotta grading system C + D grades), rates of postoperative mRS score ≤ 2, and rates of increase in mRS scores postoperatively.

Distal anterior circulation aneurysms were defined as aneurysms located in the anterior cerebral artery, middle cerebral artery, and anterior communicating artery (7).

Posterior circulation aneurysms were defined as aneurysms located in the main trunk of the vertebral-basilar artery and their branches, mainly including aneurysms in the vertebral artery, basilar artery, posterior cerebral artery, superior cerebellar artery, anterior inferior cerebellar artery, and posterior inferior cerebellar artery (8).

Ischemic complications were defined as symptomatic ischemia due to the target aneurysm and treatment-related ischemia, and they included both temporary and permanent complications throughout the postoperative follow-up period.

Intracranial complications were defined as treatment-related intracranial hemorrhages confirmed by imaging. In-stent stenosis (ISS) was defined as a 10% reduction in the diameter of the artery at the location of installation of the stent or arterial occlusion within 5 mm of either end of the stent (9).

### 2.2 Perioperative management and follow-up

Patients with unruptured aneurysms routinely received conventional dual anti-treatment (aspirin 100 mg QD + clopidogrel 75 mg QD) for 5–7 days preoperatively, and the effectiveness of the drug therapy was monitored using thromboelastography. Regarding patients who did not achieve anti-poly status, their drug dosage was adjusted based on the results of genetic monitoring, or they received aspirin 100 mg QD + ticagrelor 90 mg BID antiplatelet therapy until the target was reached. For patients with ruptured aneurysms, intravenous tirofiban was administered after intraoperative FD release (10 μg/kg by push and 0.5 μg/kg/h by continuous pumping for 12 h postoperatively). A loading dose of an oral dual antiplatelet aggregation drug (aspirin 300 mg + clopidogrel 300 mg) was administered 4 h before the end of tirofiban administration, followed by continuous dual antiplatelet aggregation drug (aspirin 100 mg QD + clopidogrel 75 mg QD) treatment. Patients were monitored for ischemic or hemorrhagic complications, and if any of these occurred, the drug dosage was promptly adjusted. Patients were examined using thromboelastography before discharge, and if arachidonic acid (AA) and adenosine phosphate (ADP) levels did not reach the standard levels (AA >50% and ADP >30%), clopidogrel was replaced with ticagrelor (90 mg/d BID). A dual antiplatelet therapy regimen was administered for 6 months postoperatively. Subsequently, patients were readmitted to the hospital for a review, and based on DSA results, a decision was made to transition to a monotherapeutic approach.

### 2.3 Statistical analysis

These analyses were performed using SPSS v25 (IBM, Armonk, NY, United States) software and the R language (version 4.2.2). The normality of continuous data was assessed using the Kolmogorov–Smirnov test. Normally distributed continuous variables are presented as mean ± standard deviation ( $\bar{x}$ ± s), and an independent sample *t*-test was used to compare group differences. Non-normally distributed data are presented as median with interquartile range (IQR) and Q50 [Q25, Q75], and the Mann–Whitney U-test was used to compare group differences. Categorical variables are presented as rates or ratios, and group differences were compared using the chi-square test or Fisher’s exact probability method. Statistical significance was set at *p* < 0.05.

Propensity score matching (PSM) was performed using the MatchIt package in R v4.2.2.^1^ Baseline data were analyzed using univariate logistic regression, and variables with statistical significance corresponding to *p* < 0.1 were included as covariates, which were integrated based on their clinical significance. A non-replacement nearest-neighbor matching with a caliper value of 0.1 was performed using a 1:1 ratio. We considered a standardized mean difference of less than 0.20 as a well-matched balance between matched cohorts after propensity score matching. Statistical analyses of the resulting matched data were performed to explore differences between the two groups.

## 3 Results

### 3.1 Baseline information

During the study period, 229 patients (246 aneurysms) and 206 patients (215 aneurysms) were enrolled in the on- and off-label groups, respectively. Patients with incomplete baseline data, incomplete imaging data, or low compliance were excluded according to the predetermined inclusion and exclusion criteria. Therefore, 242 patients with 261 aneurysms (160 in the on-label group and 101 in the off-label group) were included in the final analyses. Table 1 shows the baseline data for both groups. The reasons for selecting the 101 aneurysms in the off-label group were as follows: 92 were located in non-internal carotid arteries, 10 had acute rupture, 4 had a history of stent placement at the target aneurysm site, 16 were enrolled because of the diameter of the aneurysm-carrying artery, and 21 were selected based on their morphology. None of the aneurysms were selected based on the patients’ age. The median maximum aneurysm diameter was 4.73 [3.55, 6.33] mm and 5.23 [3.80, 6.71] mm in the on- and off-label groups, respectively. The median follow-up time for DSA was 7.13 months and 7.27 months in the on-label and off-label groups, respectively (*p* = 0.863).

**Table 1:** Baseline information in the on-label group and off-label group.

		On-label group	Off-label group	*P*-value
Baseline information	Number of patients	146	96	
	Age	54.56(52.83–56.29)	54.94(52.62–57.26)	*p* = 0.794
	Sex (female)	121/146	56/96	*P* < 0.001*
Comorbidity	Smoking	11	20	*p* = 0.003*
	Drinking	12	19	*p* = 0.008*
Hypertension	N	90	44	*p* = 0.101
	Level 1	26	23	
	Level 2	17	15	
	Level 3	15	16	
	Diabetes mellitus	10	12	*p* = 0.140
	Hyperlipidemia	54	38	*p* = 0.716
	History of cardiovascular disease	12	5	*p* = 0.363
	History of stroke/TIA	10	11	*p* = 0.220
	History of intracerebral hemorrhage	6	9	*p* = 0.100
	History of cerebral aneurysm	4	7	*p* = 0.182
	Homocysteine	11.79(10.20–13.70)	13.1(11.48–15.68)	*P* < 0.001*
Aneurysm-related	Number of aneurysms	160	101	
	Maximum diameter	4.73(3.55–6.33)	5.23(3.80–6.71)	*p* = 0.223
	Rupture	0	10	<0.001*
	History of stenting at target location	0	4	*p* = 0.043*
Location of the aneurysm	ICA	160	9	
	Anterior cerebral artery aneurysm	0	8	
	Middle cerebral artery aneurysm	0	63	
	Anterior communicating artery aneurysm	0	5	
	Basilar artery aneurysm	0	2	
	Vertebral artery aneurysm	0	12	
	PICA	0	2	
Surgery-related	Stent implantation	89	63	*p* = 0.550
	Stent-assisted embolization	59	29	
	Stent implantation+ sacculus	8	6	
	PED+embolization+sacculus	4	3	
Morphology	Fusiform/saccular	115	80	*p* = 0.184
	Other	45	21	
Dual antiplatelet therapy	Aspirin + Clopidogrel	95	59	*p* = 0.587
	Aspirin + Ticagrelor	51	37	
Preoperative mRS score	0	30	20	*p* = 0.779
	1	91	56	
	2	16	16	
	3	2	1	
	4	6	2	
	5	1	1	

TIA, transient ischemic attack; ICA, internal carotid artery; PICA, posterior inferior cerebellar artery; PED, pipeline embolization device.

Univariate analysis showed differences in baseline data between the two groups. To balance the differences between the groups, sex, smoking habit, alcohol consumption, hypertension, history of intracranial hemorrhage, and homocysteine levels at admission were matched as covariates for propensity scores, which yielded 81 matched pairs. The baseline data after matching are presented in Table 2, and the safety and efficacy outcomes are presented in Table 3. Furthermore, we used an on-label group as a control to evaluate the safety and effectiveness of PED treatment for distal anterior circulation aneurysms. Based on the results of the univariate analysis, sex, smoking habit, alcohol consumption, hypertension, history of stroke/transient ischemic attack, and homocysteine levels at admission were used as covariates for PSM. Baseline data before and after matching are presented in Supplementary Table S1, and the safety and efficacy outcomes are presented in Supplementary Table S2.

**Table 2:** Baseline information in the on-label group and off-label group after propensity score matching.

		On-label group	Off-label group	*P*-value
Baseline information	Number of patients	81	81	
	Age	55.77 ± 10.58(53.43, 58.11)	54.9 ± 11.50(52.36, 57.44)	*p* = 0.619
	Sex (female)	59(72.8%)	54(66.7%)	*p* = 0.392
Comorbidity	Smoking	8(9.9%)	12(14.8%)	*p* = 0.339
	Drinking	9(11.1%)	11(13.6%)	*p* = 0.633
	Hypertension			*p* = 0.793
	N	36(44.4%)	42(51.9%)	
	Level 1	20(24.7%)	18(22.2%)	
	Level 2	13(16%)	12(14.8%)	
	Level 3	12(14.8%)	9(11.1%)	
	Diabetes mellitus	4(4.9%)	11(13.6%)	*p* = 0.058
	Hyperlipidemia	37(45.7%)	30(37%)	*p* = 0.264
	History of cardiovascular disease	10(12.3%)	3(3.7%)	*p* = 0.079
	History of stroke/TIA	8(9%)	8(9%)	*p* = 1.00
	History of intracerebral hemorrhage	5(6.2%)	4(4.9%)	*P* = 1.00
	History of cerebral aneurysm	3(3.7%)	6(7.4%)	*p* = 0.493
	Homocysteine	12.66(11.09, 14.88)	12.97(11.41, 14.36)	*P* = 0.988
Aneurysm-related	Number of aneurysms	81	81	
	Maximum diameter	4.51(3.29, 6.21)	4.65(3.32, 7.11)	*p* = 0.807
	Rupture	0	8(9.9%)	*p* = 0.011*
	History of target aneurysm stent placement.	0	4(4.9%)	*P* = 0.129
Location of the aneurysm	ICA	81	9	
	Anterior cerebral artery aneurysm	0	7	
	Middle cerebral artery aneurysm	0	49	
	Anterior communicating artery aneurysm	0	4	
	Vertebral artery aneurysm	0	11	
	Basilar artery aneurysm	0	1	
	PICA	0	0	
Surgery-related	Stent implantation	48(59.3%)	52(64.2%)	*p* = 0.839
	Stent-assisted embolization	26(32.1%)	22(27.2%)	
	Stent implantation+Sacculus	6(7.4%)	5(6.2%)	
Number of stents	PED+Embolization+Sacculus 1 2	1 (1.2%) 81(100%) 0	2(2.5%) 78(96.3%) 3(3.7%)	*p* = 0.244
Morphology of aneurysm	Fusiform/saccular	57(70.4%)	65(80.2%)	*p* = 0.145
	Other	24(29.6%)	16(19.8%)	
Preoperative mRS score	0	13(16%)	16(19.8%)	*P* = 0.486
	1	54(66.7%)	46(56.8%)	
	2	10(12.3%)	15(18.5%)	
	3	0(0%)	1(1.2%)	
	4	4(4.9%)	2(2.5%)	
	5	0(0%)	1(1.2%)	

TIA, transient ischemic attack; ICA, internal carotid artery; PICA, posterior inferior cerebellar artery; PED, pipeline embolization device.

**Table 3:** Follow-up information in the on-label group and off-label group after propensity score matching.

		On-label group	Off-label group	*P*-value
Follow-up period		7.13(6.1, 9.48)	7.27(5.92, 10.72)	*P* = 0.863
Safety outcome	Ischemic complications	5(6.2%)	6(7.4%)	*P* = 0.755
	Intracranial hemorrhagic complications	0(0%)	0(0%)	*P* = 1.00
Procedural-related complications	Access-related complications	0(0%)	4(4.9%)	*P* = 0.129
	Complications of thrombosis	1(1.2%)	1(1.2%)	*P* = 1.00
	Vascular spasm	1(1.2%)	2(2.5%)	*P* = 1.00
	Complications related to contrast agent	0(0%)	0(0%)	*P* = 1.00
	Complications related to anesthesia	1(1.2%)	1(1.2%)	*P* = 1.00
	Other	1(1.2%)	2(2.5%)	*P* = 1.00
Efficacy outcome	Aneurysm occlusion rate	60(74.1%)	63(77.8%)	*P* = 0.581
	In-stent stenosis	4(4.9%)	17(21%)	*P* = 0.002*
	Retreatment rate in 12 months	1(1.2%)	1(1.2%)	*P* = 1.00
	Rate of increase in mRS score	2(2.5%)	3(3.7%)	*P* = 1.00
	Postoperative mRS score			*p* = 0.870
	0	45(55.6%)	45(55.6%)	
	1	27(33.3%)	28(34.6%)	
	2	6(7.4%)	6(7.4%)	
	3	3(3.7%)	1(1.2%)	
	4	0(0%)	1(1.2%)	

### 3.2 Safety outcomes

Safety outcomes were compared between the on- and off-label groups, and their results were as follows, respectively: ischemic complications, 6.2% vs. 7.4% (*p* = 0.755); thrombotic complications, 1.2% vs. 1.2% (*p* = 1.0); vasospasm, 1.2% vs. 2.5% (*p* = 1.0); anesthesia-related complications, 1.2% vs. 1.2% (*p* = 1.0); access-site complications, 0% vs. 4.9% (*p* = 0.129); and other complications, 1.2% vs. 2.5% (*p* = 1.0). No fatalities, intracranial hemorrhage complications, or contrast-related complications were reported in either group after PSM.

### 3.3 Efficacy outcomes

Efficacy outcomes were compared between the on- and off-label groups, and the results were as follows, respectively: immediate postoperative aneurysm occlusion rate, 4.9% vs. 7.4% (*p* = 0.514); aneurysm occlusion rate at follow-up, 74.1% vs. 77.8% (*p* = 0.581); rate of increased mRS scores during the follow-up period, 2.5% vs. 3.7% (*p* = 0.65); and retreatment rate within 12 months, 1.2% vs. 1.2% (*p* = 1.0). However, the incidence of in-stent stenosis (ISS) was significantly higher in the off-label group than in the on-label group (4.9% vs. 21%, *p* = 0.002).

**Figure 1:**
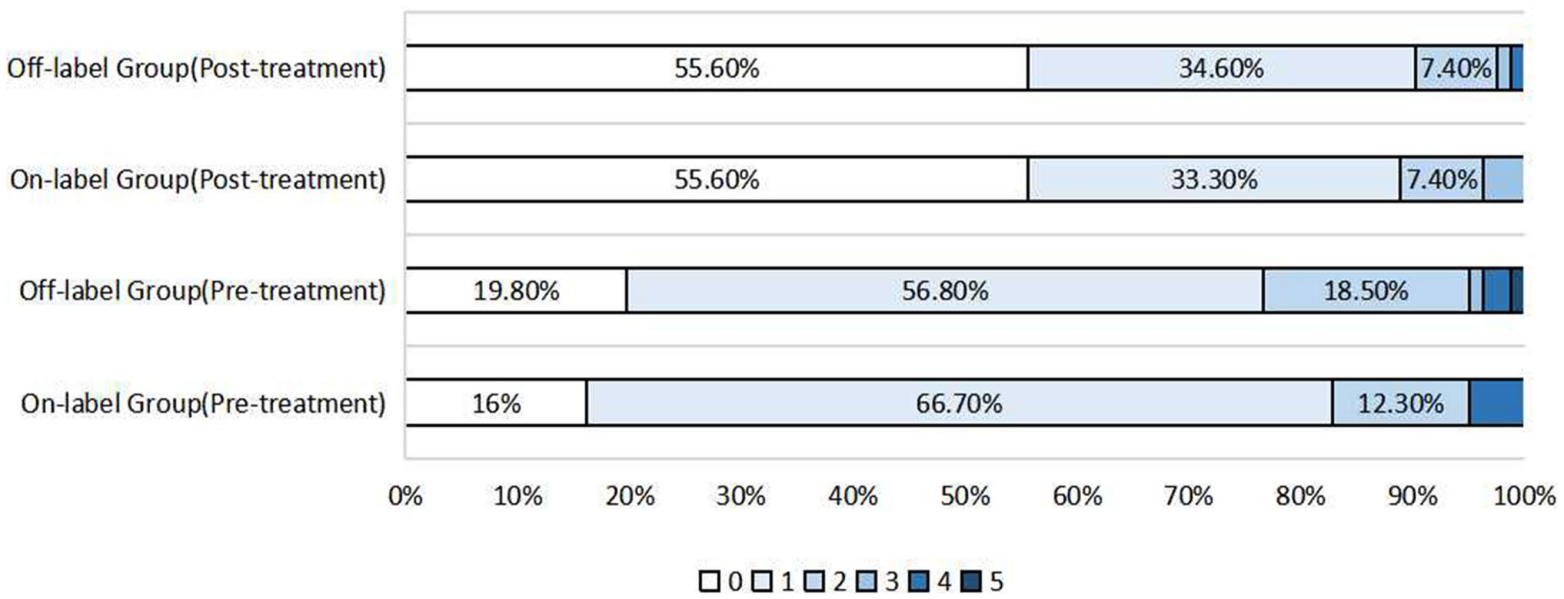
Distribution of pre-treatment mRS scores and post-treatment mRS scores after propensity score matching in the on-label group and off-label group.

**Figure 2:**
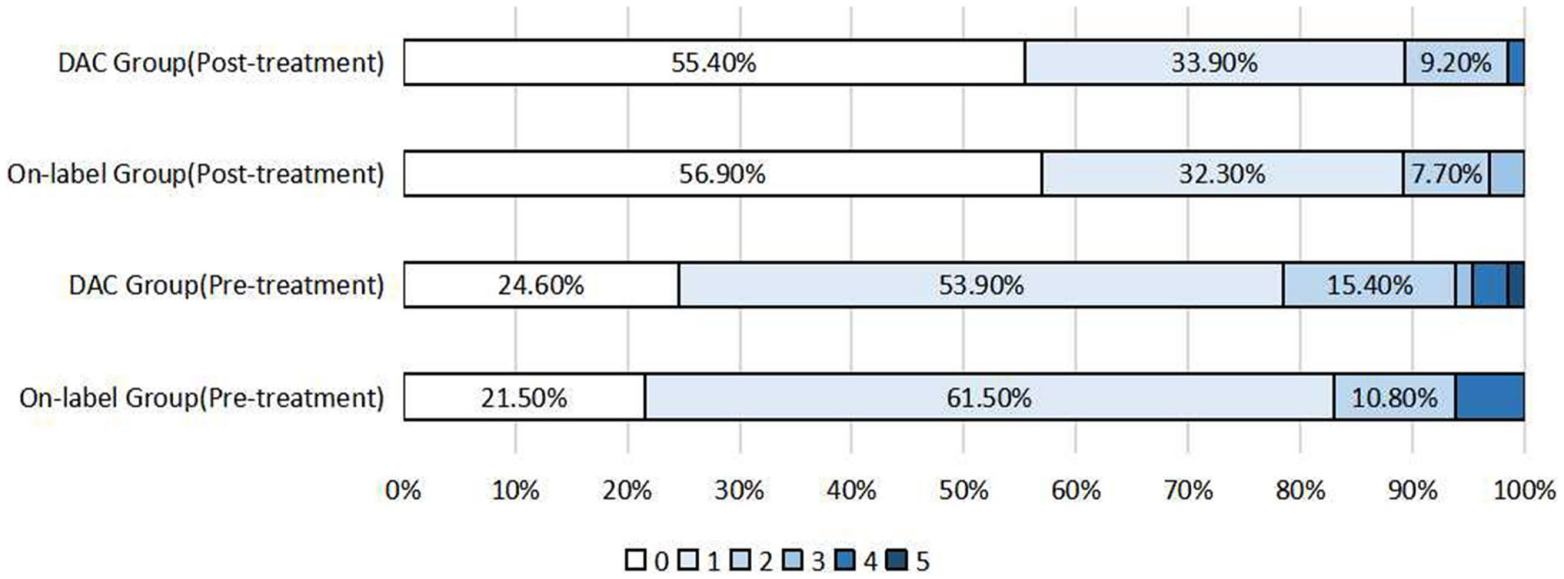
Distribution of pre-treatment mRS scores and postoperative mRS scores after propensity score matching in the on-label group and DAC group (distal anterior circulation group).

No significant difference was observed in postoperative mRS scores in 242 patients who underwent follow-up (146 in the on-label group and 96 in the off-label group) (*p* = 0.054). Furthermore, compared with the preoperative rate, the rate of postoperative favorable prognoses (mRS score ≤ 2) increased in both groups (on-label vs. off-label: 96.3% vs. 97.5%). In addition, no statistically significant differences in the distribution of postoperative mRS scores were observed between the post-PSM-adapted on-label and off-label groups (*p* = 0.486), and on-label and distal anterior circulation groups (*p* = 0.723), as shown in Figures 1, 2.

### 3.4 Subgroup analysis

#### 3.4.1 Distal anterior circulation groups

Safety outcomes were compared between on-label and the distal anterior circulation groups, and the results were as follows, respectively: ischemic complications, 4.6% vs. 7.7% (*p* = 0.715); thrombotic complications, 1.5% vs. 1.5% (*p* = 1.0); vasospasm, 0% vs. 3.1% (*p* = 0.476); anesthesia-related complications, 1.5% vs. 1.5% (*p* = 1.0); contrast-related complications, 0% vs. 1.5% (*p* = 1.0); access-site complications, 0% vs. 6.2% (*p* = 0.128); and other complications, 1.5% vs. 3.1% (*p* = 1.0). No fatalities or hemorrhagic complications were reported in either of the groups.

**Figure 3:**
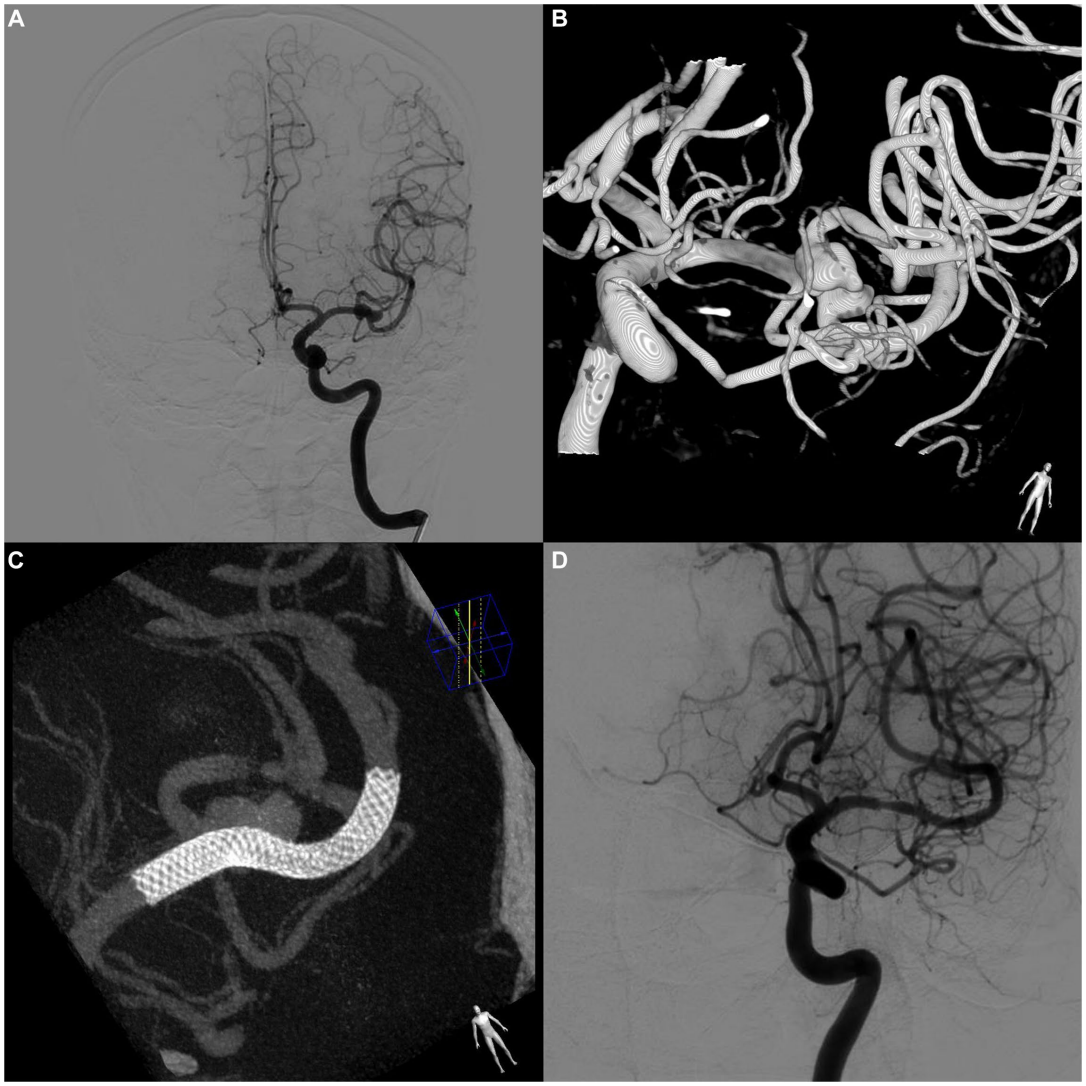
A case of asymptomatic in-stent stenosis after PED treatment of a bifurcation aneurysm. **(A)** Preoperative digital subtraction angiography (DSA) shows an aneurysm located at the bifurcation of the left middle cerebral artery. **(B)** 3D reconstructed image of the aneurysm. **(C)** DynCT shows complete stent apposition. **(D)** Follow-up DSA at 8 months postoperatively shows complete occlusion of the aneurysm. The stenosis within the stent at the beginning of the stent was approximately 35%. No relevant clinical symptoms were observed.

Efficacy outcomes were compared between the on-label group and the distal anterior circulation group, and the results were as follows, respectively: immediate postoperative aneurysm occlusion rate, 6.2% vs. 6.2% (*p* = 1.0); aneurysm occlusion rate at follow-up, 75.4% vs. 81.5% (*p* = 0.393); rate of increased mRS scores during the follow-up period, 1.5% vs. 1.5% (*p* = 1.0); and retreatment rate within 12 months, 1.5% vs. 0% (*p* = 1.0). Furthermore, the incidence of ISS significantly differed between the two groups (7.7% vs. 24.6%, *p* = 0.009). A typical case is shown in Figure 3.

#### 3.4.2 Posterior circulation aneurysms

There were 16 cases of posterior circulation aneurysms, with an average aneurysm size of 7 mm and a median follow-up period of 7.37 months. The occlusion rate was 62.5, and 31.25% of aneurysm sacs decreased in size. One patient (6.25%) developed a femoral artery pseudoaneurysm, and another (6.25%) experienced transient dizziness after the procedure. One patient (6.25%) with a basilar artery aneurysm experienced a recurrence and required additional treatment. No deaths, intracranial hemorrhage complications, or permanent complications occurred. The rate of favorable outcomes was 93.75%.

#### 3.4.3 Recurrent aneurysms

There were four cases of recurrent aneurysms, with a median follow-up of 7 months. The occlusion rate was 75%. One patient had stent thrombosis postoperatively. No intracranial hemorrhages or permanent complications occurred. The rate of favorable outcomes was 100%.

#### 3.4.4 Ruptured aneurysms

Moreover, there were 10 cases of ruptured aneurysms, with a mean aneurysm size of 8.86 mm and a median follow-up of 7 months. The occlusion rate was 80%. One patient developed a femoral pseudoaneurysm after the procedure, and another patient who had a bifurcation aneurysm in the middle cerebral artery underwent a cerebral ventricular shunt for vasospasm and hydrocephalus (mRS score of 4). Nine patients had favorable outcomes (90%) without intracranial hemorrhage complications, aneurysm re-rupture, or permanent adverse events.

#### 3.4.5 In-stent stenosis

In the present study, ISS was defined as a 10% decrease in the arterial diameter of a stented segment or an arterial occlusion within 5 mm of the stent ends, which differed from the previous definition of a 25% decrease in arterial diameter (10, 11). The incidence of ISS after the implantation of PEDs was 25% (4/16), 21.1% (16/76), and 7.5% (12/160) in the posterior circulation aneurysm, distal anterior circulation aneurysm, and on-label groups, respectively. There was no statistically significant difference between the distal anterior and posterior circulation groups (*p* = 0.988). After PSM, the incidence of ISS was higher in the off-label group than in the on-label group (21% vs. 4.9%, *p* = 0.002). Similarly, it was higher in the distal anterior circulation group than in the on-label group (24.6% vs. 7.7%, *p* = 0.009). All 16 patients with distal anterior circulation and 4 with posterior circulation were asymptomatic.

## 4 Discussion

In this study, we investigated the safety and efficacy of the off-label use of PEDs for treating intracranial aneurysms and found that overall clinical complications, prognoses, and imaging outcomes were comparable between the on- and off-label groups. The incidence of ISS was higher in the off-label group; however, all patients were asymptomatic and did not require clinical intervention. Because each group varied in terms of aneurysm location and vascular condition, refraining from generalizing is important. Compared with the surgical clipping procedure, PED implantation for aneurysms provides several notable advantages. First, it reduces surgical complexity and duration, which decreases the risk of complications in patients with poor surgical tolerance (12, 13). Second, it allows for less intervention in the aneurysm because the device is positioned inside the parent artery. Third, the mechanical damage inflicted on the aneurysm wall is significantly minimized during the surgical procedure compared with that in coil embolization. Fourth, it significantly reduces the incidence of common space-occupying effects resulting from coil embolization. Consequently, even for aneurysms requiring immediate protection through coil embolization, PED implantation may reduce the number of required coils, potentially downsizing the aneurysmal sac volume. Studies have shown that PED implantation can reduce fluoroscopy time, decrease contrast agent dose, and protect patients and surgical operators to a certain extent compared with traditional coil embolization surgery (14). However, there is no consensus on the safety and efficacy of the off-label use of PEDs, both domestically and abroad. Therefore, exploring the safety and efficacy of PEDs for such aneurysms is paramount to providing a substantial theoretical foundation for making clinical decisions.

### 4.1 Aneurysm occlusion rate

The overall rate of aneurysm occlusion was 81% during the follow-up period of 7.2 months. Notably, 122 of 160 aneurysms in the on-label group were successfully occluded, resulting in an occlusion rate of 83.6%. In the off-label group, 80 of 101 aneurysms were occluded, resulting in an occlusion rate of 79.2%. The occlusion rate of distal anterior circulation aneurysms was 84.7%, whereas that of posterior circulation aneurysms was 62.5% (10/16). After PSM, no statistically significant difference in the occlusion rates of aneurysms was observed between the off- and on-label groups or between the on-label and distal anterior circulation groups. However, the occlusion rate of posterior circulation aneurysms was lower than that of anterior circulation aneurysms. Notably, most of the posterior circulation aneurysms with poor imaging results (83.3%, 5/6) had associated branching vessels, and one (16.7%) had a history of stent placement at the target aneurysm site. Both factors are predictors of a lower likelihood of aneurysm occlusion (13, 14). Previous studies have reported significant differences in imaging results and clinical functional scores owing to variations in morphological and size characteristics among the included aneurysms (15–19). Further studies are necessary to validate the efficacy of PED therapy for posterior circulation aneurysms.

Though Cagnazzo et al. proposed that the location of middle cerebral arteries is an independent risk factor for aneurysm non-occlusion (20), in the present study, we yielded similar occlusion rates for on-label and distal anterior circulation aneurysms after PED treatment, which constituted 84.6% of middle cerebral artery aneurysms and had an overall occlusion rate of approximately 80%. The overall rate of aneurysm occlusion in our study is a little lower than that of the PLUS study (88.9% in a mean follow-up period of 9.6 months), which may contribute to the shorter follow-up period in our study. However, the occlusion rate in aneurysms that are located at or beyond the circle of Willis is higher in our study (84.7% in our study and 62.9% in PLUS) (21). Given the high occlusion rate of distal anterior circulation aneurysms in this study, we suggest that PED may be an effective treatment for these aneurysms.

### 4.2 Incidence of complications

No significant differences in the incidence of complications or postoperative clinical functional scores were observed among the on-label, off-label, and distal anterior circulation groups. However, the off-label and distal anterior circulation groups exhibited higher complication rates at the entry site (4.9 and 6.2%, respectively) than the indication group. This may be attributed to the intricate vascular conditions, relatively narrow diameter of the aneurysmal artery, and insufficient support at the distal end, leading to prolonged surgical time and complex instrument manipulation, resulting in corresponding complications.

Previous studies have suggested that posterior circulation aneurysms may have a higher complication rate (22, 23). However, no serious ischemic events occurred in the 16 cases of posterior circulation aneurysms included in the present study, possibly because of the strict administration of antiplatelet therapy before and after surgery.

### 4.3 ISS rate

ISS is a common outcome after stent implantation. According to a recent study, PED-induced intimal hyperplasia reaches its pinnacle within 1 year and resolves within 2 years (22). Chalouhi et al. reported that during an average follow-up period of 6.7 months, 15.8% (*n* = 22) of patients developed stenosis within the stent, with 11 patients developing mild stenosis and 11 developing moderate-to-severe stenosis (>50%). Notably, all patients were asymptomatic. The authors suggested that ISS was more likely to occur after PED use for internal carotid artery aneurysms (16.7%) than for anterior circulation aneurysms (7.6%) (23). This finding was inconsistent with the results reported by Gui et al., who revealed a higher incidence of ISS after PED use for posterior circulation aneurysms. In the present study, no significant difference in the incidence of stenosis within stents was observed in patients with anterior and posterior circulation distal aneurysms; however, the off-label (*p* = 0.002) and distal anterior circulation (*p* = 0.009) groups demonstrated a higher incidence of stenosis within stents compared with the on-label group. The incidence of stenosis within stents was reported to be 11.4 and 25% in patients with anterior and posterior circulation distal aneurysms, respectively. This may seem higher than the in-stent stenosis rate reported in the PLUS study (10.03%). However, it is worth noting that the majority of aneurysms in the PLUS study were located in the internal carotid artery, and the definition of ISS in PLUS was a lumen diameter loss of >50%, which is quite different from ours (24). Strictures that develop within stents after implantation have been identified as benign anatomical changes that result from intravascular intimal hyperplasia. Patients with such stenoses generally remain asymptomatic and may exhibit a degree of improvement in imaging over a certain follow-up period (10, 22). Therefore, excessive clinical intervention to manage this condition may be unnecessary. Animal studies have shown that PED implantation may alleviate the incidence of strictures within stents by curtailing intimal hyperplasia without affecting the aneurysm occlusion rate (25).

This study had some limitations. First, the study was a retrospective analysis. PSM was used to balance baseline differences between the two groups; however, some unmeasured biases could still exist. Second, imaging data were obtained only from symptomatic patients; therefore, patients with asymptomatic ischemia or bleeding were excluded from this study. Third, imaging data were primarily obtained during hospitalization, and short-term neurological complications that occurred outside the hospital may have been overlooked, potentially leading to an underestimation of the complication rate. Fourth, the median follow-up period in this study was relatively short, potentially leading to an underestimation of the occlusion rate. Fifth, long-term imaging information regarding stenosis within the stent was unavailable. Finally, the study groups were only classified based on FDA standards, and further stratification by aneurysm morphology, size, or location was not performed, which may have resulted in bias in the study results.

## 5 Conclusion

The off-label use of PED to treat intracranial aneurysms did not increase the incidence of symptomatic complications or the rate of retreatment compared with on-label use, and the neurological and imaging outcomes of on- and off-label indications were similar. Therefore, decisions regarding clinical management should not rely solely on on- or off-label indications. Physicians’ experience and comprehensive postoperative management may be critical to the prognosis of aneurysms. Furthermore, prospective studies with larger sample sizes are required to investigate the safety and efficacy of PEDs for posterior circulation aneurysms.

## Data availability statement

The raw data supporting the conclusions of this article will be made available by the authors, without undue reservation.

## Ethics statement

The studies involving humans were approved by the Ethics Committee of Scientific Research and Clinical Trail of the First Affiliated Hospital of Zhengzhou University, ethics number: KY 2018–098-02. The studies were conducted in accordance with the local legislation and institutional requirements. Written informed consent for participation was not required from the participants or the participants’ legal guardians/next of kin in accordance with the national legislation and institutional requirements.

## Author contributions

YM: Data curation, Formal analysis, Methodology, Writing – original draft. XD: Data curation, Supervision, Writing – original draft. ZC: Data curation, Formal analysis, Writing – original draft. YY: Data curation, Formal analysis, Writing – original draft. SG: Conceptualization, Methodology, Supervision, Validation, Writing – review & editing. XG: Conceptualization, Methodology, Supervision, Validation, Writing – review & editing.

## Footnotes

^1^ www.r-project.org
