# Supplementary material for: Safety and efficacy analysis of the off-label use of pipeline embolization devices for intracranial aneurysms: a propensity score matching study
Source: Front Neurol. 2024 Jan 4;14:1278366. doi: 10.3389/fneur.2023.1278366 (PMC10794508; doi:10.3389/fneur.2023.1278366)
Supplement: Supplementary file 1 [file Data_Sheet_1.docx]

Supplemental Table 1. Baseline information in distal anterior circulation group and on-label group

|  |  | Before PSM | |  | After PSM | |  |
| --- | --- | --- | --- | --- | --- | --- | --- |
|  |  | On-label group | Distal anterior circulation group | P-value | On-label group | Distal anterior circulation group | P-value |
| Baseline information | Number of Patients | 146 | 72 |  | 65 | 65 |  |
|  | Age | 54.57±10.56(52.84,56.30) | 56.69±11.10(54.09,59.30) | P=0.171 | 56.86±10.10(54.36,59.36) | 57.06±10.83(54.38,59.74) | P=0.913 |
|  | Sex(female) | 121 | 45 | P=0.001 | 50 | 46 | P=0.425 |
| Comorbidity | Smoking | 11 | 13 | P=0.020 | 10 | 8 | P=0.612 |
|  | Drinking | 11 | 13 | P=0.020 | 10 | 8 | P=0.612 |
|  | Hypertension | 57 | 43 | P=0.004 | 39 | 36 | P=0.594 |
|  | Diabetes mellitus | 10 | 9 | P=0.164 | 4 | 8 | P=0.226 |
|  | Hyperlipidemia | 52 | 24 | P=0.739 | 29 | 20 | P=0.103 |
|  | History of cardiovascular disease | 12 | 3 | P=0.408 | 7 | 3 | P=0.188 |
|  | History of stroke/TIA | 10 | 10 | P=0.090 | 7 | 7 | P=1.00 |
|  | History of intracerebral hemorrhage | 6 | 6 | P=0.332 | 3 | 5 | P=0.715 |
|  | History of cerebral aneurysm | 4 | 3 | P=0.878 | 2 | 2 | P=1.0 |
|  | Homocysteine | 11.70(10.19,13.68) | 13.04(11.45,15.63) | P=0.001 | 12.56(11.50,14.38) | 12.71(11.41,14.40) | P=0.948 |
| Aneurysm-related | Number of aneurysms | 160 | 76 |  | 65 | 65 |  |
|  | Maximum diameter | 4.70(3.46,7.82) | 4.24(2.80,6.23) | P=0.131 | 4.67(3.46,7.40) | 4.22(2.86,6.21) | P=0.294 |
|  | Rupture | 0 | 2 | P=0.108 | 0 | 2 | P=0.476 |
|  | History of target aneurysm stent placement | 0 | 1 | P=0.330 | 0 | 1 | P=1.00 |
| Location of the aneurysm | ICA | 160 | 0 |  | 65 | 0 |  |
|  | Anterior communicating artery aneurysm | 0 | 4 |  | 0 | 4 |  |
|  | Anterior cerebral artery aneurysm | 0 | 9 |  | 0 | 6 |  |
|  | Middle cerebral artery aneurysm | 0 | 63 |  | 0 | 55 |  |
| Surgery-related | Stent implantation | 80 | 47 | P=0.559 | 31 | 45 | P=0.052 |
|  | Stent-assisted embolization | 55 | 21 |  | 26 | 17 |  |
|  | Stent implantation+Sacculus | 7 | 3 |  | 7 | 2 |  |
|  | PED+Embolization+Sacculus | 4 | 1 |  | 1 | 1 |  |
|  | Fusiform/saccular | 115 | 61 | P=0.167 | 45 | 53 | P=0.103 |
| Morphology of aneurysm | Other | 45 | 15 |  | 20 | 12 |  |
| Pre-operative mRS score | 0 | 30 | 17 | P=0.734 | 14 | 16 | P=0.664 |
|  | 1 | 90 | 39 |  | 40 | 35 |  |
|  | 2 | 16 | 12 |  | 7 | 10 |  |
|  | 3 | 3 | 1 |  | 0 | 1 |  |
|  | 4 | 6 | 2 |  | 4 | 2 |  |
|  | 5 | 1 | 1 |  | 0 | 1 |  |

Supplemental Table 2. Follow-up information in distal anterior circulation group and on-label group

|  |  | Before PSM | |  | After PSM | |  |
| --- | --- | --- | --- | --- | --- | --- | --- |
|  |  | On-label group | Distal anterior circulation group | P-value | On-label group | Distal anterior circulation group | P-value |
|  | Follow-up period | 6.93(6.09,9.43) | 7.17(5.81,11.18) | P=0.841 | 7(6.1,10.1) | 7.27(6.03,11.15) | P=0.805 |
| Safety outcome | Ischemic complications | 10(6.3%) | 6(7.9%) | P=0.639 | 3（4.6%） | 5（7.7%） | P=0.715 |
|  | Intracranial hemorrhagic complications | 1(0.6%) | 0(0%) | P=1.00 | 0(0%) | 0(0%) | P=1.00 |
| Procedural-related complications | Access-related complications | 1(0.6%) | 4(5.3%) | P=0.068 | 0（0%） | 4(6.2%) | P=0.128 |
|  | Complications of thrombosis | 3(1.9.%) | 1(1.3%) | P=1.00 | 1(1.5%) | 1(1.5%) | P=1.00 |
|  | Vascular spasm | 1(0.6%) | 2(2.6%) | P=0.243 | 0(0%) | 2(3.1%) | P=0.476 |
|  | Complications related to contrast agent | 2(1.3%) | 1(1.3%) | P=1.00 | 0(0%) | 1(1.5%) | P=1.00 |
|  | Complications related to anesthesia | 1(0.6%) | 1(1.3%) | P=0.541 | 1(1.5%) | 1(1.5%) | P=1.00 |
|  | Other | 7(4.4%) | 2(2.6%) | P=0.722 | 1(1.5%) | 2(3.1%) | P=1.00 |
| Efficacy outcome | Aneurysm occlusion rate | 132（82.5%） | 63（82.9%） | P=0.940 | 49(75.4%) | 53(81.5%) | P=0.393 |
|  | In-stent stenosis | 12(7.5%) | 16(21.1%) | P=0.003 | 5(7.7%) | 16(24.6%) | P=0.009 |
|  | Retreatment rate in 12months | 1(0.6%) | 0(0.0%) | P=1.00 | 1(1.5%) | 0(0%) | P=1.00 |
|  | Rate of increase in mRS score | 3(2.1%) | 1(1.4%) | P=1.00 | 1(1.5%) | 1(1.5%) | P=1.00 |
|  | Postoperative mRS score |  |  | P=0.557 |  |  | P=0.723 |
|  | 0 | 87 | 42 |  | 37 | 36 |  |
|  | 1 | 43 | 23 |  | 21 | 22 |  |
|  | 2 | 13 | 6 |  | 5 | 6 |  |
|  | 3 | 3 | 0 |  | 2 | 0 |  |
|  | 4 | 0 | 1 |  | 0 | 1 |  |
